# Pulsed Electromagnetic Field Promotes Bone Anabolism in Postmenopausal Osteoporosis through the miR-6976/BMP/Smad4 Axis

**DOI:** 10.1155/2023/8857436

**Published:** 2023-06-03

**Authors:** Jinming Huang, Yi Li, Siyi Zhu, Liqiong Wang, Hongliang Pei, Xiangxiu Wang, Tianjie Bao, Zhiyuan Jiang, Lin Yang, Chengqi He

**Affiliations:** ^1^Department of Rehabilitation Medicine, West China Hospital, Sichuan University, Chengdu, Sichuan 610041, China; ^2^Key Laboratory of Rehabilitation Medicine, West China Hospital, Sichuan University, Chengdu, Sichuan 610041, China; ^3^Human Engineering Laboratory, The School of Mechanical Engineering, Sichuan University, Chengdu, Sichuan 610041, China; ^4^Department of Plastic Surgery, Sichuan Provincial People's Hospital, University of Electronic Science and Technology of China, Chengdu, Sichuan 610072, China

## Abstract

**Background:**

Insufficient bone formation is the key reason for the imbalance of bone metabolism and one of the main mechanisms for the occurrence and deterioration of postmenopausal osteoporosis (PMOP). Accumulating evidence has demonstrated that pulsed electromagnetic field (PEMF), as a physiotherapy, can treat osteoporosis by promoting osteogenic differentiation in osteoblasts. However, little is known about its mechanisms.

**Methods:**

*In vivo*, ovariectomized mice were administered PEMF for 4 weeks, and skeletal analysis was conducted. *In vitro*, hydrogen peroxide-treated mouse osteoblast precursor cells with or without PEMF intervention were subjected to osteogenic differentiation testing and miRNA microarrays. The potential target miRNAs were validated, followed by gene expression assays to further clarify their regulatory relationships with target pathways.

**Results:**

We found that PEMF reduced bone loss in ovariectomized mice and promoted osteogenic differentiation of hydrogen peroxide-treated osteoblast precursor cells via downregulation of miR-6976-5p. Mechanistically, reduced miR-6976-5p enhanced the nuclear transport of phosphorylated Smad1/5/9 by upregulating Smad4, thereby activating the BMP/Smad pathway. Additionally, the administration of miR-6976-5p inhibitors successfully promoted osteogenic differentiation in vitro, and its antagomirs protected bone mass in vivo. miR-6976-5p mimics and agomirs acted in the opposite way.

**Conclusion:**

These results provide evidence that PEMF alleviates estrogen deficiency-induced bone loss by activating osteoblastic progenitor cells and maintaining their osteogenic differentiation and shed light on the mechanisms involved, which may provide a potential option for the clinical application of PEMF in PMOP.

## 1. Introduction

Osteoporosis is a systemic skeletal disease characterized by decreased bone mass and degenerated bone microarchitecture, accompanied by increased bone fragility and risk of bone fracture, among which postmenopausal osteoporosis (PMOP) is the most common [1, 2]. In recent years, the prevalence of PMOP and the incidence of fragility fractures have been increasing [3, 4]. Approximately, 50% of postmenopausal women experience at least one bone fracture [5]. Bone mass loss and microstructure deterioration occur when the rate of osteoblast-driven bone formation fails to reach that of osteoclast-driven bone resorption, and women with estrogen deficiency suffer from this pathological process [1, 6]. Therefore, promoting bone formation shows great potential in the prevention and treatment of PMOP [7, 8].

Pulsed electromagnetic field (PEMF) is a physical therapy that is a characteristic short wave with a specific signal shape and low frequency (5–300 Hz) generated by alternating current through two or more external electromagnetic coils [9]. Since 1979, PEMF has been approved by the U.S. Food and Drug Administration (FDA) for the treatment of ununited fractures and failed arthrodeses for its advantages of noninvasiveness, low cost, and few adverse events [10]. Studies have confirmed that PEMF promotes fracture healing through multiple signaling pathways, including the BMP/Smad pathway, Wnt/*β*-catenin pathway, Notch/NICD pathway, JAK-STAT pathway, mTOR pathway, PTH/MAPK/ERK pathway, and VEGF pathway [11]. Recently, accumulating evidence suggests that PEMF is also effective in PMOP: PEMF is as effective as alendronate in maintaining bone mass [12], and it can effectively prevent bone loss, reduce pain, and improve patient outcomes [13–15] without specific adverse events [16]. However, the mechanism by which PEMF treats PMOP is not fully understood, which limits its application.

The BMP/Smad pathway is one of the key pathways for osteogenesis. Smad4, the unique common Smad (Co-Smad), is an essential component of the BMP/Smad pathway and is the only cofactor of receptor-regulated Smads (R-Smads, including Smad 1/2/3/5/8/9) [17, 18]. Smad4 plays an important role in stem cell differentiation, stemness maintenance, and self-renewal in osteogenic progenitor cells [19, 20]. Specifically, Smad4 regulates the balance of MSC lineage commitment by modulating the retention of Taz in the nucleus in osteogenic and adipogenic differentiation pathways [21]. Smad4 directly binds to regulatory elements in the *Runx2* promoter, thereby upregulating Runx2 expression to support bone and cartilage development [22]. In addition, Smad4 is required to inhibit osteoclastogenesis and maintain bone mass [23].

Nevertheless, the contribution of Smad4 to PEMF-promoted osteogenic differentiation remains unresolved. More importantly, the molecular mechanisms governing Smad4 are not clear.

Accumulating evidence has revealed that microRNA (miRNA)-mediated posttranscriptional regulation may coordinate transcription factors to determine cell fate [24]. miRNAs have been reported to regulate the differentiation of osteogenic progenitor cells and the process of bone formation [25–27]. However, it is still unknown whether miRNAs also contribute to PEMF-promoted osteogenic differentiation. Here, we report that decreased miR-6976-5p induced by PEMF targets *Smad4*, regulates the osteogenic differentiation of MC3T3-E1 cells, and reduces bone loss in ovariectomized (OVX) mice.

## 2. Materials and Methods

### 2.1. Animal Experiments

All animal experimental procedures were approved by the Animal Ethics Committee of West China Hospital of Sichuan University (No. 2021022A). Sixty 8-week-old C57/BL female mice (HFK Bioscience, China) were used for the study. They were maintained under standard conditions (12 h light/dark cycle, temperature 20–22°C and 60% humidity) and fed standard pellets and purified water ad libitum at the Laboratory Animal Center. After 4 weeks of acclimation, mice were randomized into 3 groups with 12 mice in each group: OVX group, OVX + PEMF group, and sham-operation group (SHAM). Periovarian adipose tissue, which was similar in size to the ovaries, was removed in the SHAM group, while bilateral OVX was performed in the other groups. All treatments were executed 1 week after OVX and lasted for 4 weeks (Figure S1A).

miRNA agomiR or antagomiR (GenePharma, China) delivery in mice was conducted after OVX. We injected these RNA oligos intravenously via mouse tail veins at a dose of 10 mg/kg of the body weight in 0.1 ml saline once a week until sampling. Details of the agomiR and antagomiR sequences of miRNA-6976-5p are shown in Table S2.

### 2.2. Cell Culture and Osteogenic Differentiation

Mouse preosteoblastic MC3T3-E1 cells (CAT. GDC0188) were purchased from the China Center for Type Culture Collection (Wuhan, China) and were cultured in alpha-MEM containing 10% FBS and 1% penicillin and streptomycin. The osteogenic medium (OS) consisted of basal growth medium with 10 mM *β*-glycerophosphate, 50 ng/mL L-ascorbic acid, and 10 nM dexamethasone (Sigma‒Aldrich, USA). The cells were seeded at a density of 2 × 10^4^ cells/cm^2^ until reaching 80% confluency. Then, we treated the cells with 400 *μ*M H_2_O_2_ for 4 h to induce oxidative stress and performed subsequent experiments.

### 2.3. PEMF Treatment

The PEMF device for cells was placed in an incubator, and the device for animals was placed in a mouse breeding room (FiguresS1A and S1B). The PEMF stimulation parameter was based on our previous data: square wave/1.6 mT/75 Hz. [28–30]. We used a handheld Gaussian meter (HT201; Hengtong, China) to measure the magnetic field intensity. Details of the PEMF exposure device can be found in our previous studies [28].

### 2.4. Microcomputed Tomography (Micro-CT)

We used a micro-CT scanner (Quantum GX; Germany) to scan the distal femurs with scanning time of 14 minutes, pixel size of 20 *μ*m, X-ray energy 90 kV, and current intensity of 88 *μ*A. The corresponding analysis software (PerkinElmer Analyze 12.0) was applied to get imaging parameters, including trabecular thickness (Tb.Th), trabecular number (Tb.N), trabecular spacing (Tb.Sp), bone volume fraction (BV/TV), and bone surface/bone volume ratio (BS/BV). The area of interest was the 50 layers below the epiphyseal plate.

### 2.5. Histology and Immunohistochemistry

Mouse femurs were decalcified with 10% EDTA, dehydrated with increasing concentrations of ethanol, embedded in paraffin, then sliced into sagittal sections (5-mm-thick) for dewaxing, washing, and H and E staining. Multinucleated osteoclasts were labeled by a tartrate-resistant acid phosphatase (TRAP) kit (Sigma–Aldrich, USA), after which these cells located in the metaphyseal region were counted.

The methodological details of immunohistochemistry staining can be found in our previous study [28].

### 2.6. Osteogenic Efficiency Detection

Western blotting was performed to detect protein expression. Protein extracts were separated by 8%–15% SDS-polyacrylamide gels and transferred to PVDF membranes. Membranes were blocked with 5% nonfat milk for 1 h and then incubated with the indicated antibodies (HuaBio, China) overnight at 4°C. On the next day, these membranes were washed with TBST 4 times for 5 min each time. Then, antigen-antibody reactions were visualized by enhanced chemiluminescence assay (Bio-Rad) after incubation with HRP-conjugated secondary antibody for 1 h at 25°C. Subcellular fraction proteins were prepared using a Nuclear Protein Extraction Kit (Solarbio, China). ImageJ software was used to quantify the intensity of grayscale images.

We used real-time PCR to determine the mRNA levels of osteogenesis-related genes (Runx2, Osx, etc.) [28]. The primer sequences are shown in Table S1

Detection of bone mineralization and osteogenic activity: cells were fixed with 4% paraformaldehyde at 25°C for 30 min, washed three times with PBS, stained with Alizarin Red Stain (ARS) solution (Cyagen, China) at room temperature, protected from light for 10 min, washed three more times, and photographed under a microscope. After that, 10% cetylpyridine ammonium chloride solution was added to each well and placed on a shaker for half an hour, and then the absorbance was measured at OD570. Osteogenic activity was detected and quantified using an alkaline phosphatase (ALP) assay kit (Nanjing Jiancheng, China) according to the instructions.

### 2.7. Cell Transfections, Silencing, and Overexpression

We seeded MC3T3-E1 cells to be 60–80% confluent. The next day, we diluted Lipofectamine® RNAiMAX Reagent into alpha-MEM medium and miRNA mimics/inhibitors (GenePharma, China) or small interfering RNA (siRNA) (Tsingke, China) to 50 nm in alpha-MEM medium. Then, we added the latter to the former reagent and incubated it for 5 minutes at room temperature. Finally, the recommended dose of the complex was added to the medium of cells according to the instructions. We analyzed those cells over the next 24–72 hours. Details of the miRNA mimics/inhibitors and siRNA sequences are shown in Table S2.

### 2.8. Dual-Luciferase Reporter Assay

Wild-type (WT) or mutant (Mut) 3′UTRs of *Smad4* containing its predicted miRNA-binding sites were synthesized and cloned into the pEZX-FR02 (Genecopoeia) plasmid, whose sequences already included endogenous controls (Figure S2). We seeded 293T cells to be 50–60% confluent. On the second day, cotransfection was performed using the JetPRIME@transfection reagent (Polyplus, France). 50 *μ*g Smad4-WT/Mut plasmid and 50 nm miR-6976-5p mimics/NC were simultaneously diluted in 200 *μ*l JetPRIME@buffer, vortexed for 10 s, added 2 *μ*l JetPRIME@, mixed slowly, and incubated at room temperature for 10 min. Then, 200 *μ*l of transfection mixture was added to each well. Replace fresh medium after 4 h of cotransfection. Cells were lysed 24 h after cotransfection, and luciferase activities were measured by a dual-luciferase assay system (Vazyme, DL 101-01, China) according to the instruments.

### 2.9. Immunofluorescence Staining

Immunofluorescence staining was performed on cell climbing slices. The fixed cells were permeabilized with 0.1% Triton X − 100 for 10 mins at 25°C, blocked with 5% BSA for 1 h at 37°C, and incubated with anti-Smad4 antibody (1 : 50) overnight at 4°C. Then, slices were immunostained with Alexa Fluor 488-conjugated secondary antibodies (Jackson ImmunoResearch, USA) for 1 h at 37°C, then stained with DAPI, and finally photographed with an upright fluorescence microscope (Nikon, Japan).

### 2.10. miRNA Sequencing and Prediction of miRNA Targets

Total RNA from H_2_O_2_-induced MC3T3-E1 cells with or without PEMF exposure was collected in TRIzol reagent (TAKARA. Japan). We assessed RNA quantity and purity before sequencing. RIN value ≥ 7 indicated acceptable RNA integrity, and A260/A280 ≥ 1.5 and A260/A230 ≥ 1.0 indicated acceptable RNA purity. miRNA-seq sequencing and analysis were performed by Guangzhou Huayin Health Medical Group Co., Ltd (Guangzhou, China), who used an illumina instrument (NovaSeq 6000 Sequencing system) for miRNA sequencing and several databases for analysis (**supplementary material appendix 3**).

We used miRNA target prediction algorithms, including TargetScan, miRanda, and Pictar, to identify the potential targets of miRNAs with significant differences detected by miRNA sequencing. All three databases predicted Smad4 as the target gene of miR-6975-5p.

### 2.11. Statistics

We used GraphPad Prism software for statistical analysis and graphing. The data are presented as the mean ± SD. One-way ANOVA and the Kruskal‒Wallis tests were applied to compare multiple groups for data conforming to the normal distribution and non-normal distribution, respectively. Tukey's test and Dunn's test were used to perform post-hoc multiple testing for normally distributed data and non-normally distributed data, respectively. The statistical significance threshold was set at *P* < 0.05 (two-tailed).

## 3. Results

### 3.1. PEMF Reduced Bone Loss in Ovariectomized Mice

In agreement with previous studies, we observed that OVX was accompanied by rapid destruction of the bone microstructure, including attenuation of the growth plate, reduction in bone mass, and decrease in various remodeling parameters.

**Figure 1:**
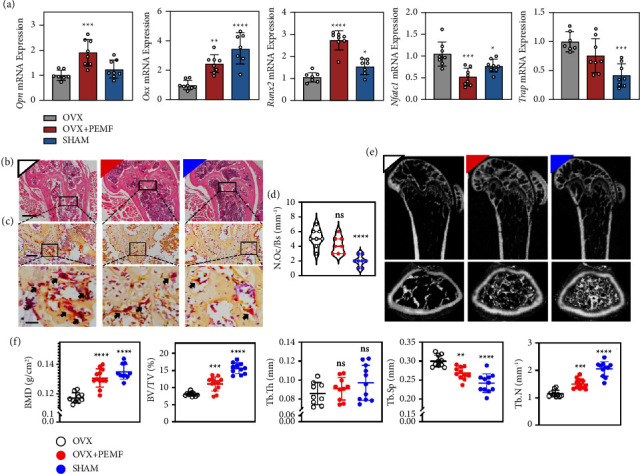
PEMF protects the microstructure of the distal femur in ovariectomized (OVX) mice. (a) Relative mRNA expression of Opn, Osx, Runx2, Nfctc1, and Trap (*n* = 6–8 independent experiments). (b) Representative H and E staining of femoral sections; scale bar = 500 *μ*m. (c) Images of TRAP-positive multinucleated osteoclasts (indicated by arrows); scale bar = 100 and 20 *μ*m for the top and bottom images, respectively. (d) Calculated osteoclast number from (c) (*n* = 8 per group). (e) 2D and 3D reconstruction images obtained by micro-CT show the details of trabecular bones in distal femurs. (f) Quantitative parameters of micro-CT images including BMD, BV/TV, Tb.Th, Tb.Sp, and Tb.N (*n* = 9–12 per group). All scatter plot data are the mean ± SD. ^*∗*^*P* < 0.05, ^*∗∗*^*P* < 0.01, ^*∗∗*^*P* < 0.001, ^*∗∗∗*^*P* < 0.0001.

First, the PCR results (Figure 1(a), Figure S1C) showed that the expression levels of osteogenic markers (*Runx2, Opn,* and *Col1a*) in the distal femur specimens of the PEMF treatment group were significantly higher than those of the OVX group. Meanwhile, the osteoclast-related factor nuclear factor of activated T cells 1 (Nfatc1) was slightly decreased after intervention, suggesting that PEMF may promote bone formation.

Next, the mouse distal femur specimens were subjected to pathological and imaging analyses to investigate the protective effect of different interventions. H and E staining (Figure 1(b)) indicated that the femoral epiphyseal plates of OVX mice were significantly thinner than those of the SHAM group, while the thickness of the epiphyseal plates of the PEMF group was similar to that of the SHAM group. TRAP staining (Figures 1(c) and 1(d)) showed that PEMF treatment blocked the increase in osteoclasts to some extent. Micro-CT 2D and 3D reconstructed images (Figure 1(e)) revealed that the PEMF group rescued OVX-induced bone microarchitectural disruption and the bone loss phenotype. Based on these images, quantitative analyses (Figure 1(f)) showed that the bone volume fraction (BV/TV) of the PEMF group increased by 37.55% compared with that of the OVX group. In addition, bone mineral density (BMD), trabecular thickness (Tb.Th), trabecular number (Tb.N), and trabecular separation (Tb.Sp) values were all restored after PEMF intervention. A similar trend was obtained by 4.2 immunohistochemical staining for Opn and Bmp2 (Figure S1D).

### 3.2. PEMF Promoted Osteogenic Differentiation in MC3T3-E1 Cells by Activating the BMP/Smad Pathway

**Figure 2:**
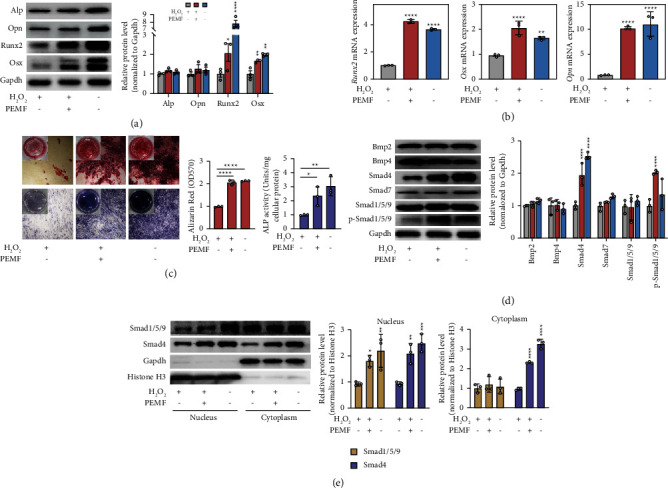
PEMF promotes osteogenic differentiation and activates the BMP/Smad pathway. (a) Western blotting and quantitative data of osteogenic markers in different groups (*n* = 3 independent experiments). (b) Relative mRNA expression of Runx2, Osx, and Opn was measured by qRT-PCR (*n* = 3 independent experiments). (c) ARS (top) and ALP staining (bottom) were used to evaluate calcium deposition and osteogenic activity, respectively (*n* = 3 independent experiments). (d) Western blotting and quantitative data of the BMP/Smad pathway (*n* = 3 independent experiments). (e) Western blotting and quantitative data of the subcellular localization of Smad1/5/9 and Smad4 from nuclear and cytosolic extracts. GAPDH and histone H3 were used as the cytosolic extract loading control and the nuclear extract loading control, respectively. (*n* = 3 independent experiments). ^*∗*^*P* < 0.05, ^*∗*^*P* < 0.01, ^*∗∗∗*^*P* < 0.001, ^*∗∗∗∗*^*P* < 0.0001.

In vitro, we observed similar results to in vivo experiments. The protein (Figure 2(a)) and mRNA (Figure 2(b)) levels of osteogenic markers (Opn, Runx2, and Osx) in H_2_O_2_-treated MC3T3-E1 cells were obviously elevated after PEMF intervention. Notably, the mRNA expression of *Runx2* and *Osx* was even slightly higher in the H_2_O_2_ + PEMF group than in the blank group. ARS staining (Figure 2(c)) showed that the H_2_O_2_ + PEMF group had more calcium deposits and more regularly packed collagen fibrils than the H_2_O_2_ group. Consistently, ALP staining showed a similar trend of osteogenic activity as ARS. The above results suggest that PEMF can promote bone formation in oxidatively stressed MC3T3-E1 cells. In order to investigate the mechanisms of PEMF- facilitated osteogenic differentiation in MC3T3-E1 cells, we detected the activation of the BMP/Smad signaling pathway, which is one of the key pathways of bone formation [31, 32]. Compared with H_2_0_2_-treated MC3T3-E1 cells, we found that BMP/Smad signaling was activated in normal MC3T3-E1 cells as determined by the upregulation of phosphorylated R-Smads, which are downstream of this cascade (Figure 2(d)). Correspondingly, Smad4 was significantly upregulated. However, Smad7 remained unchanged. Interestingly, PEMF intervention did not alter the expression of the upstream signals but elevated the expression of Smad4 and the phosphorylation levels of R-Smads (Figure 2(d), S1E), resulting in increased nuclear translocation of R-Smads, as determined by Western blotting analysis of subcellular fractions (Figure 2(e)).

### 3.3. PEMF Alters miRNA Expression Profiles during Osteogenic Differentiation

**Figure 3:**
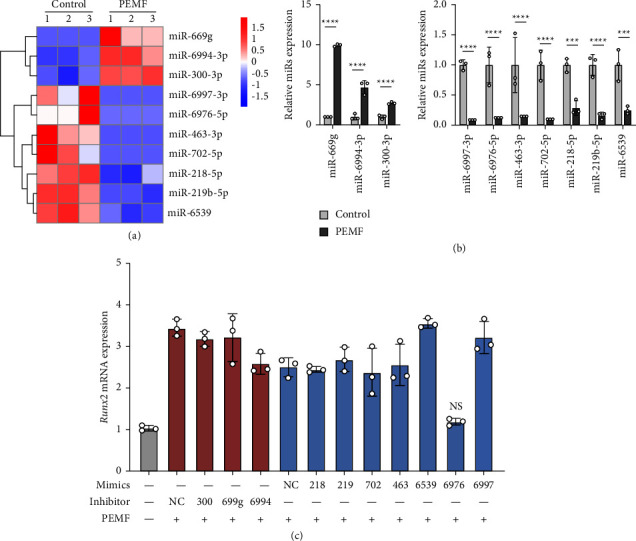
PEMF alters miRNA expression profiles in osteogenic differentiation of H_2_0_2_-treated MC3T3-E1 cells. (a) Microarray analysis was used to detect the miRNA expression profiles of H_2_O_2_-induced MC3T3-E1 cells with or without PEMF intervention. (*n* = 3 independent experiments). Shown in the heatmap are predicted miRNAs targeting the BMP/Smad pathway. (b) Relative expression of the indicated miRNAs in MC3T3-E1 cells treated as in (a) determined by qRT-PCR. (c) Relative mRNA expression of Runx2 in H_2_O_2_-treated MC3T3-E1 cells in different groups. ^*∗*^*P* < 0.05, ^*∗∗*^*P* < 0.01, ^*∗∗∗*^*P* < 0.001, ^*∗∗∗∗*^*P* < 0.0001; NS: no significance between the indicated groups.

To investigate whether miRNAs are related to the process of PEMF-promoted osteogenic differentiation, we carried on miRNA sequencing to compare the miRNA expression in H_2_O_2_-treated MC3T3-E1 cells (control group) and cells treated with PEMF intervention (PEMF group). The criteria for screening differentially expressed miRNAs was fold change >2.5 or <0.4. We filtered out the miRNAs that predicted targeting the BMP/Smad pathway and selected the 10 miRNAs with the top *p* values for further study (Figure 3(a)). qRT-PCR was used to validate changes in these miRNAs (Figure 3(b)).

### 3.4. miR-6976-5p Regulates the Osteogenic Differentiation of MC3T3-E1 Cells

To investigate the role of the above miRNAs in the osteogenic differentiation of H_2_O_2_-treated MC3T3-E1 cells, we transduced PEMF-treated MC3T3-E1 cells with miRNA mimics for each downregulated miRNA and miRNA inhibitors for each upregulated miRNA. Considering that Runx2 is a critical transcription factor in bone formation [33] and previous results have shown that Runx2 has good specificity, we detected the expression of *Runx2* after transduction with miRNA mimics/inhibitors. We observed that cells transduced with only miR-6976-5p mimics reduced *Runx2* expression after PEMF treatment (Figure 3(c)).

**Figure 4:**
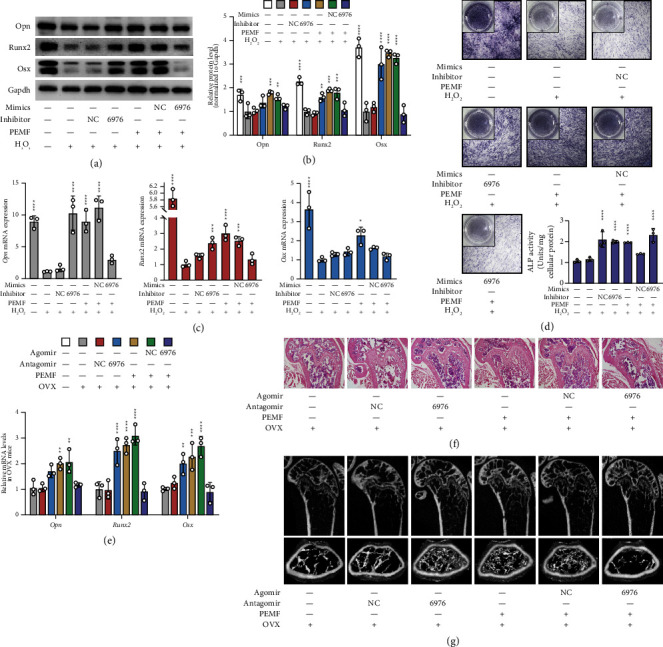
miR-6976-5p regulates osteogenic differentiation of H_2_O_2_-treated MC3T3-E1 cells. (a-b) H2O2-treated MC3T3-E1 cells were transfected with miR-6976 inhibitor or inhibitor NC or miR-6976 mimic or mimic NC and treated with or without PEMF for 72 h. The protein levels of Opn, Runx2, and Osx were determined by western blotting followed by quantitative analysis (n = 3 independent experiments). (c) Relative expression of osteogenic markers in MC3T3-E1 cells treated as in (a) determined by qRT-PCR (n = 3 independent experiments). (d) The osteogenic activity of cells treated as in (a) was evaluated by ALP staining (n = 3 independent experiments). (e) The OVX mice were administered miR-6976 antagomir or antagomir NC or miR-6976 agomir or agomir NC and treated with or without PEMF for 4 weeks. The mRNA expression levels of Opn, Runx2, and Osx were determined by qRT-PCR (n = 3 independent experiments). (f) H&E staining of femoral sections of mice treated as in (e). (g) 2D and 3D reconstruction images obtained by micro-CT show the details of trabecular bones in distal femurs of mice treated as in (e). ^*∗*^*P* < 0.05, ^*∗∗*^*P* < 0.01, ^*∗∗*^*P* < 0.001, ^*∗∗∗*^*P* < 0.0001.

To further verify the roles of miR-6976-5p in osteogenic differentiation, we transduced H_2_O_2_-treated MC3T3-E1 cells with miR-6976-5p inhibitors and transduced PEMF-treated cells with miR-6976-5p mimics. As shown in Figures 4(a), 4(b), and 4(d), the expression of osteogenic markers was detected by western blotting (Figures 4(a) and 4(b)) and qRT-PCR (Figure 4(c)**)**. H_2_O_2_-treated cells transduced with miR-6976-5p inhibitors expressed more Opn, Runx2, and Osx than those without transduction. Meanwhile, the PEMF-treated MC3T3-E1 cells transduced with miR-6976-5p mimics expressed fewer osteogenic factors than those without transduction. Consistently, ALP staining showed higher ALP activity in the induced osteoblasts transduced with miR-6976-5p inhibitors, and lower ALP activity in the PEMF-treated cells transduced with miR-6976-5p mimics, which almost entirely abrogated the positive effects of PEMF in boosting oxidative damaged cell osteogenic differentiation (Figure 4(d)).

In addition, we evaluated the effects of miR-6976-5p on bone formation in OVX mice. As shown in Figure 4(e), qRT-PCR indicated that osteogenic markers were upregulated in both the OVX + miR-6976-5p antagomir group and OVX + PEMF group compared with the OVX group, while these markers were downregulated in the OVX + PEMF + miR-6976-5p agomir group. Correspondingly, the results of H and E staining and micro-CT 3D reconstruction images also indicated that the miR-6976-5p antagomir imitated the osteogenic effects of PEMF, and the miR-6976 agomir abrogated the positive effects of PEMF (Figures 4(f) and 4(g)).

### 3.5. miR-6976-5p Inhibits Osteogenic Differentiation by Targeting Smad4 Both *In Vivo* and *In Vitro*

**Figure 5:**
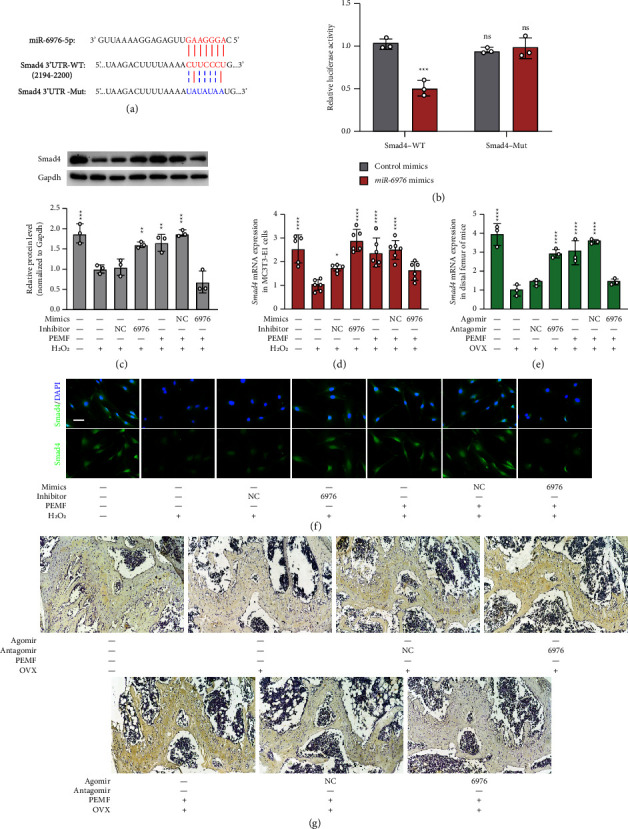
miR-6976-5p targets Smad4. (a) The complementary pairing of miR-6976-5p with *Smad4*wild-type (WT) and mutant (Mut) 3′UTR reporter constructs is shown. (b) The reporter plasmids pEZX-FR02 carrying the WT or Mut Smad4 3′UTR regions were cotransfected with miR-6976-5p mimic or mimic NC into 293T cells. The dual luciferase reporter assays were performed after 24 h (*n* = 3 independent experiments). (c) H_2_O_2_-treated MC3T3-E1 cells were transfected with miR-6976 inhibitor or inhibitor NC or miR-6976 mimic or mimic NC and treated with or without PEMF for 72 h the protein levels of Smad4 were determined by western blotting followed by quantitative analysis (*n* = 3 independent experiments). (d) The mRNA expression of *Smad4* in cells treated as in (c) determined by qRT-PCR (*n* = 3 independent experiments). (e) The OVX mice were administered miR-6976 antagomir or antagomir NC or miR-6976 agomir or agomir NC and treated with or without PEMF for 4 weeks. The mRNA expression level of *Smad4* was determined by qRT-PCR(*n* = 3 independent experiments). (f) MC3T3-E1 cells traded as (c) subjected to Smad4/DAPI immunofluorescent staining after 48 h (*n* = 3 independent experiments). Scale bar = 50 *μ*m. (g) Immunohistochemical images show the relative protein expression of Smad4 in the metaphysis area in mice treated as in (e) (*n* = 3 independent experiments). ^*∗*^*P* < 0.05, ^*∗∗*^*P* < 0.01, ^*∗∗∗*^*P* < 0.001, ^*∗∗∗∗*^*P* < 0.0001; NS: no significance between the indicated groups.

Having established that miR-6976-5p plays a key role in osteogenesis by targeting the BMP/Smad pathway, we speculated that Smad4 mRNA 3′-UTR harbored putative miR-6976-5p binding sites via three miRNA target algorithms (miRanda, Pictar, and TargetScan). To determine whether the 3′-UTR of Smad4 is a functional target of miR-6976-5p, we cloned the Smad4 3′-UTR fragment that contained the putative binding sites into a luciferase reporter vector, pEZX-FR02 (Figure 5(a)), and cotransfected it with miR-6976-5p mimics. The luciferase activity was specifically reduced by miR-6976-5p mimics (Figure 5(b)), and this effect was completely abrogated by mutations in the miR-6976 binding sequences, confirming the specificity of the miRNA target sites (Figure 5(b)). Hence, *Smad4* is a target gene of miR-6976-5p.

Next, we evaluated whether miR-6976-5p influenced *Smad4* expression. Transduction of H_2_O_2_-treated MC3T3-E1 cells with miR-6976-5p inhibitors or tail vein injection of OVX mice with miR-6976-5p antagomir increased the expression of *Smad4* (Figures 5(c)–5(e)). Conversely, transduction of PEMF-treated cells with miR-6976-5p mimics or tail vein injection of PEMF-treated OVX mice with miR-6976-5p agomir decreased the expression of *Smad4* (Figure 5(c)–5(e)). Furthermore, we applied immunofluorescence staining and immunohistochemical staining to semiquantitate and semilocalize Smad4 in MC3T3-E1 cells (Figure 5(f)) and in the femoral diaphysis (Figure 5(g)), respectively. The trend of the results is consistent with the above results.

### 3.6. PEMF Upregulated Smad4 by Decreasing miR-6976-5p and Thus Promoting Osteogenesis

**Figure 6:**
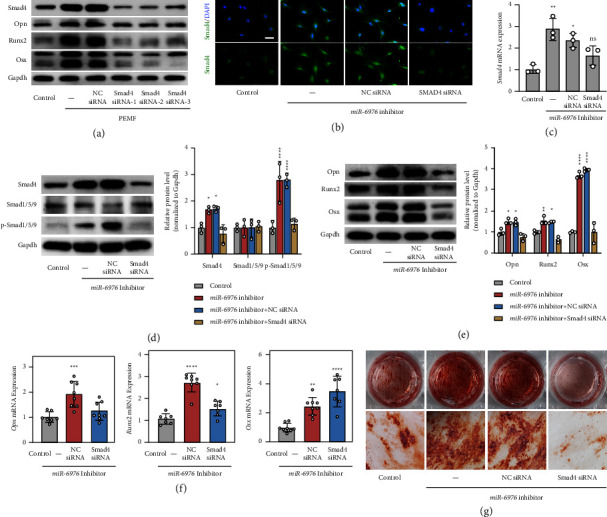
PEMF upregulated Smad4 by decreasing miR-6976-5p and thus promoting osteogenesis. (a) H_2_O_2_-treated MC3T3-E1 cells were exposed with/without PEMF and transfected with Smad4 siRNA1/2/3 or NC siRNA. Protein levels of Smad4 and osteogenic markers were detected by western blotting. (*n* = 3). (b) MC3T3-E1 cells transfected with miR-6976 inhibitor alone or cotransfected with NC siRNA or Smad4 siRNA. Immunofluorescent staining of Smad4/DAPI was performed after 48 h (*n* = 3). Scale bar = 50 *μ*m. (c) Relative mRNA expression of Smad4 in cells treated as in (b) determined by qRT-PCR (*n* = 3). (d) The protein levels of Smad4, Smad1/5/9, and phosphorylated Smad1/5/9 in cells treated as in (b) were determined by western blotting followed by quantitative analysis (*n* = 3). (e) The protein levels of Opn, Runx2, and Osx in cells treated as in (b) were determined by western blotting followed by quantitative analysis (*n* = 3). (f) Relative expression of osteogenic markers in MC3T3-E1 cells treated as in (b) determined by qRT-PCR (*n* = 3). (g) ARS was used to evaluate calcium deposition in cells treated as in (b) after 28 days (*n* = 3). ^*∗*^*P* < 0.05, ^*∗∗*^*P* < 0.01, ^*∗∗∗*^*P* < 0.001, ^*∗∗∗∗*^*P* < 0.0001.

To test whether PEMF-induced miR-6976-5p downregulation results in enhanced osteogenic differentiation by elevating *Smad4*, we interfered with *Smad4* expression in MC3T3-E1 cells by transfecting *Smad4* small interfering RNA (siRNA). Our data suggested that inhibition of *Smad4* reversed the PEMF-induced promotion of osteogenic differentiation in MC3T3-E1 cells and decreased the protein levels of osteogenic markers (Figure 6(a)). We found that the cotransfection of miR-6976-5p inhibitors and *Smad4* siRNA inhibited the expression of Smad4 (Figures 6(b)–6(d)), which suppressed the phosphorylation of R-Smads and ultimately led to BMP/Smad signaling inactivation (Figure 6(d)). It was observed that cotransfection not only resulted in lower mRNA and protein levels of Opn, Runx2, and Osx (Figures 6(e) and 6(f)) but also induced less calcium deposits (Figure 6(g)); thus, PEMF-induced positive effect in osteogenic differentiation was abrogated. These data suggest that decreased miR-6976-5p expression contributes to stronger osteogenic activity by upregulating Smad4.

## 4. Discussion

**Figure 7:**
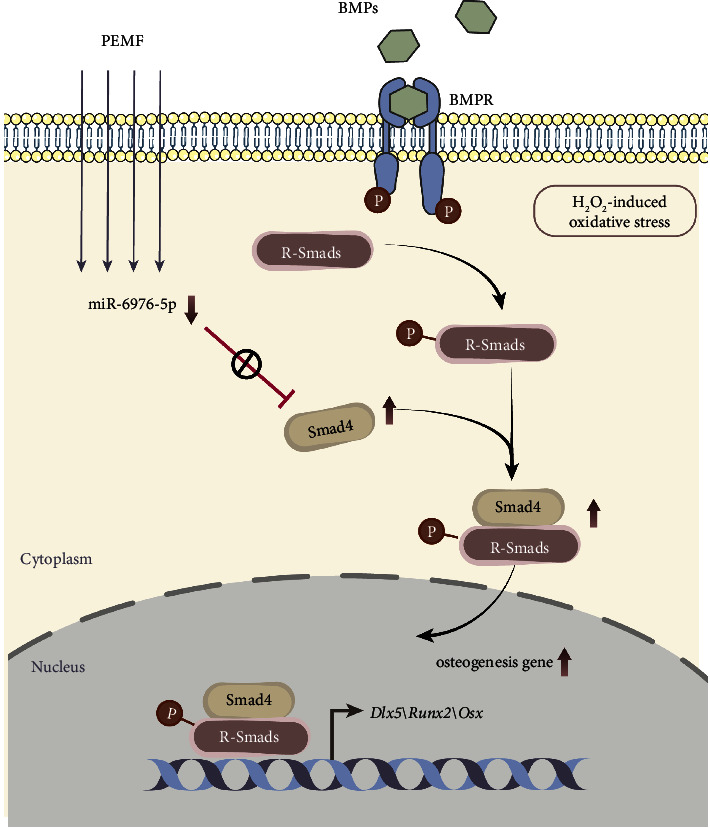
Schematic highlighting the main findings of this study. PEMF decreases miR- 6976-5p, which targets Smad4 and promotes osteogenic differentiation in H_2_O_2_-treated MC3T3-E1 cells.

PMOP is a common bone metabolic disease. Estrogen deficiency leads to a decrease in the osteogenic differentiation capacity of osteoblastic progenitor cells and a deterioration of the cellular microenvironment (e.g., oxidative stress, inflammation). Consequently, new matrix cannot fill the resorption lacunae and net bone loss ensues [8, 34–36]. At present, commonly used drugs to promote bone regeneration are romosozumab and tipapeptide. However, adverse events associated with the monoclonal antibody romosozumab include hyperosteoplasia, mandibular osteonecrosis, cardiovascular events, osteoarthritis, and cancer [37]. Teripapeptide, a derivative of parathyroid hormone, may increase the incidence of bone tumors, such as osteoma, osteoblastoma, and osteosarcoma [38]. Additionally, both are characterized by high price and a long course of treatment. These factors lead to a decrease in patients' acceptance and compliance with treatment, which makes the therapeutic effect of PMOP not as expected. Therefore, safer and more effective strategies to promote bone regeneration are urgently needed. Here, we demonstrate that PEMF reduces bone loss in a PMOP mouse model and promotes osteoblast differentiation in H_2_O_2_-treated MC3T3-E1 cells by downregulating miR-6976-5p. Mechanistically, decreased miR-6976-5p enhances R-Smads phosphorylation by upregulating *Smad4*. This study gains insights into the mechanism of osteogenic differentiation that may contribute to new strategies for the prevention and treatment of PMOP and even other metabolic bone diseases (Figure 7).

It is reported that PEMF has multiple biological functions in bone metabolism, including promoting osteogenesis, [39, 40] attenuating osteoclast activity [41, 42], inhibiting cellular inflammation [43, 44], etc. PEMF is also a novel potential physiotherapy to promote osteogenesis and has attractive traits including good safety, noninvasiveness, low cost, and easy operation [15]. Previous studies have shown that PEMF promotes bone differentiation through several signaling pathways, including the BMP signaling pathway [11, 45]. We dissected the mechanisms of this phenomenon and revealed that PEMF facilitates R-Smad phosphorylation by upregulating Smad4, a positive role in BMP/Smad signaling, thereby promoting bone formation.

R-Smads, molecules immediately downstream of BMPR, play a critical role in BMP signal transduction. Evidence shows that phosphorylation and nuclear translocation of R-Smads are positively related to bone development [32, 46]. Our data revealed that PEMF helps to maintain R-Smads phosphorylation and transfer phosphorylated R-Smads to the nucleus by upregulating its synergy factor, Smad4. Mechanistically, Smad4 and R-Smads form relatively stable complexes (Smad4/R-Smads) and synergistically enter the nucleus, activating the transcription of osteogenic factors [18, 47]. We found that Smad4 plays a major role in PEMF-promoted osteogenic differentiation. *Smad4* is targeted by miR-6976-5p, while PEMF can downregulate miR-6976-5p, thus elevating the expression level of *Smad4* and activating BMP/Smad signaling.

miRNAs are small endogenous noncoding RNAs that regulate gene expression posttranscriptionally. It is known that their regulatory effects in biological processes are based on their diverse target genes and are accomplished through multiple cellular pathways [48]. Accumulating evidence verified manipulating specific miRNAs expression can affect bone metabolism [49]. In bone formation, miRNAs play a regulatory role in cell cycle progression, differentiation commitment, and fine regulation of gene expression during osteogenic differentiation [50, 51]. Nevertheless, whether miRNAs participate in PEMF-induced osteogenesis has poorly been explored. Our study reveals that PEMF facilitates bone formation in the mouse PMOP model and osteoblasts through downregulation of miR-6976-5p. Also, miR-6976-5p antagomirs and inhibitors successfully reduced bone loss in vivo and promoted osteogenic differentiation in MC3T3-E1 cells, respectively.

Recently, several studies have indicated that PEMF manipulates osteogenic differentiation by altering the expression of some miRNAs, which is a novel strategy to modify or enhance bone formation. Selvamurugan [52] found that PEMF activated the TGF-*β*-Smad pathway to promote hBMSCs differentiation by increasing miRNA-21 and downregulating its target gene *Smad7* (an inhibitory Smad). Monica [53] found that PEMF could increase miR-26a and miR-29b and decrease miR-125b. They believed that these miRNAs might promote osteogenesis by regulating osteogenesis-angiogenesis coupling, but the mechanism was unclear. Our findings complement the mechanisms by which PEMF promotes osteogenesis through miRNAs.

Our study holds its own limitations. First, the directions of the magnetic field acting on the mice cannot be determined because the position of the mice in the cage is not fixed. Second, specific pulsed magnetic field parameters potentially restrict the generalizability of the present findings. Third, due to technical limitations, we failed to isolate bone marrow mesenchymal stem cells from OVX mice, so the in vitro experimental model used in our study is an oxidatively damaged pro-osteoblast cell line that is not fully representative of an in vitro model of osteoporosis.

## 5. Conclusion

With the progressive aging of the general population, PMOP has emerged as a growing public health concern and socioeconomic problem. One of the major schemes for preventing PMOP is to promote osteoblast differentiation and bone matrix synthesis, which can prevent and compensate for the deterioration of bone microstructure caused by estrogen deficiency. We demonstrated that the application of PEMF in the early stages of PMOP can restrict estrogen-induced bone loss effectively, possibly by activating the BMP/Smad4 pathway by regulating miRNAs at the pretranscriptional level. As a simple, low-cost, and effective physical therapy, PEMF may be a potential treatment for PMOP. It makes long-term health care economics sense if PEMF devices can be introduced into healthcare institutions (e.g., community service centers) to access clinical applications.

## Data Availability

The datasets used and/or analyzed during the current study are available from the corresponding authors on reasonable request.

## Supplementary Materials

Supplementary Materials Supplementary data for this article can be found in the supplementary material file. (1) Supplementary Tables. Table S1. Primer sequence information used in RT-PCR, Table S2. Sequences of oligos; (2) Supplementary Figures, Figure S1, Figure S2; (3) Database and analysis software used for microRNA sequencing.
