# Supplementary material for: Pulsed Electromagnetic Field Promotes Bone Anabolism in Postmenopausal Osteoporosis through the miR-6976/BMP/Smad4 Axis
Source: J Tissue Eng Regen Med. 2023 Jun 3;2023:8857436. doi: 10.1155/2023/8857436 (PMC11919207; doi:10.1155/2023/8857436)
Supplement: Supplementary Materials — Supplementary data for this article can be found in the supplementary material file. (1) Supplementary Tables. Table S1. Primer sequence information used in RT-PCR, Table S2. Sequences of oligos; (2) Supplementary Figures, Figure S1, Figure S2; (3) Database and analysis software used for microRNA sequencing. [file 8857436.f1.docx]

**Supplemental material**

**1. Supplementary Tables.**

**Table S1. Primer sequence information used in RT-PCR**

**Table S2. Sequences of oligos**

**2. Supplementary Figures**

**Figure S1**

**Figure S2**

**3. Database and analysis software used for microRNA sequencing**

**1. Supplementary Tables**

**Table S1. Primer sequence information used in RT-PCR**

| **Genes** | **Primers** | **Primer sequence (5’-3’)** | **Accession number** | **Product length (bp)** | **TM (℃)** |
| --- | --- | --- | --- | --- | --- |
| *COL1a* | Forward  Reverse | GCGAAGGCAACAGTCGCT  CTTGGTGGTTTTGTATTCGATGAC | NM_007742.4 | 156 | 56.2 |
| *RUNX2* | Forward  Reverse | GGTACTTCGTCAGCATCCTATCAG  GCTTCCGTCAGCGTCAACAC | NM_001145920.2 | 156 | 59.5 |
| *OPN* | Forward  Reverse | CTCCAATCGTCCCTACAGTCG  AGGTCCTCATCTGTGGCATC | NM_001204201.1 | 127 | 57.4 |
| *BMP2* | Forward  Reverse | GGAAAAGGACATCCGCTCCA  GCCACGATCCAGTCATTCCA | NM_007553.3 | 137 | 57.4 |
| *Bmp6* | Forward  Reverse | AGTACGTCCCCAAACCATGC  AGTACGTCCCCAAACCATGC | NM_007556.4 | 127 | 55.0 |
| *TRAP* | Forward  Reverse | GGCTCAAAAAGCAGTTGGCA  GTAGGCAGTGACCCCGTATG | NM_001102404.1 | 149 | 56.9 |
| *NFATc1* | Forward  Reverse | CGCAAGTACAGTCTCAATGG  CAGGTATCTTCGGTCACACT | NM_001164109.1 | 104 | 55.4 |
| *CTSK* | Forward  Reverse | AGAACGGAGGCATTGACTCT  GATGGACACAGAGATGGGTC | NM_007802.4 | 176 | 57.5 |
| *BAX* | Forward  Reverse | GATCCAAGACCAGGGTGGCT  TCTTCTTCCAGATGGTGAGCG | NM_007527.3 | 116 | 57.6 |
| *SMAD4* | Forward  Reverse | CCTGTTGTGACTGTGGAT  CCTGTGGACATTGGAGAG | NM_001364967.1 | 80 | 54.9 |
| *GAPDH* | Forward  Reverse | TGCACCACCAACTGCTTAG  GGATGCAGGGATGATGTTC | NM_001289726.1 | 177 | 55.2 |

**Table S2. Sequences of oligos**

| **Name of the oligo** | **Sequence (5’-3’)** |
| --- | --- |
| mimics/agomir negative control | UUGUACUACACAAAAGUACUG |
| inhibitors/antagomir negative control | CAGUACUUUUGUGUAGUACAA |
| mmu-miR-6976-5p mimic/agomir | CAGGGAAGUUGAGAGGAAAAUUG |
| mmu-miR-6976-5p inhibitor/antagomir | CAAUUUUCCUCUCAACUUCCCUG |
| mmu-miR-300-3p inhibitor | GAAGAGAGCUUGCCCUUGCAUA |
| mmu-miR-669g inhibitor | AUCAUGUCAACACAUACAAUGCA |
| mmu-miR-6994-3p inhibitor | GCAAAGACGGAGAAGAUCGUU |
| mmu-miR-218-5p mimics | UUGUGCUUGAUCUAACCAUGU |
| mmu-miR-219b-5p mimics | AGAUGUCCAGCCACAAUUCUCG |
| mmu-miR-702-5p mimics | GUGAGUGGGGUGGUUGGCAUG |
| mmu-miR-463-3p mimics | UGAUAGACACCAUAUAAGGUAG |
| mmu-miR-6539 mimics | GCACAGUGAUGAACUCUGAGGGCU |
| mmu-miR-6997-3p mimics | UCAAACCUUACCCUCCUGUUUCC |
| Smad4 siRNA-1-F | CAGUAUGCGUUUGACUUAATT |
| Smad4 siRNA-1-R | UUAAGUCAAACGCAUACUGTT |
| Smad4 siRNA-2-F | CUCCAUUGCUUACUUUGAATT |
| Smad4 siRNA-2-R | UUCAAAGUAAGCAAUGGAGTT |
| Smad4 siRNA-3-F | GCGACGCUGUUCAUAAGAUTT |
| Smad4 siRNA-3-R | AUCUUAUGAACAGCGUCGCTT |

**2. Supplementary Figures**


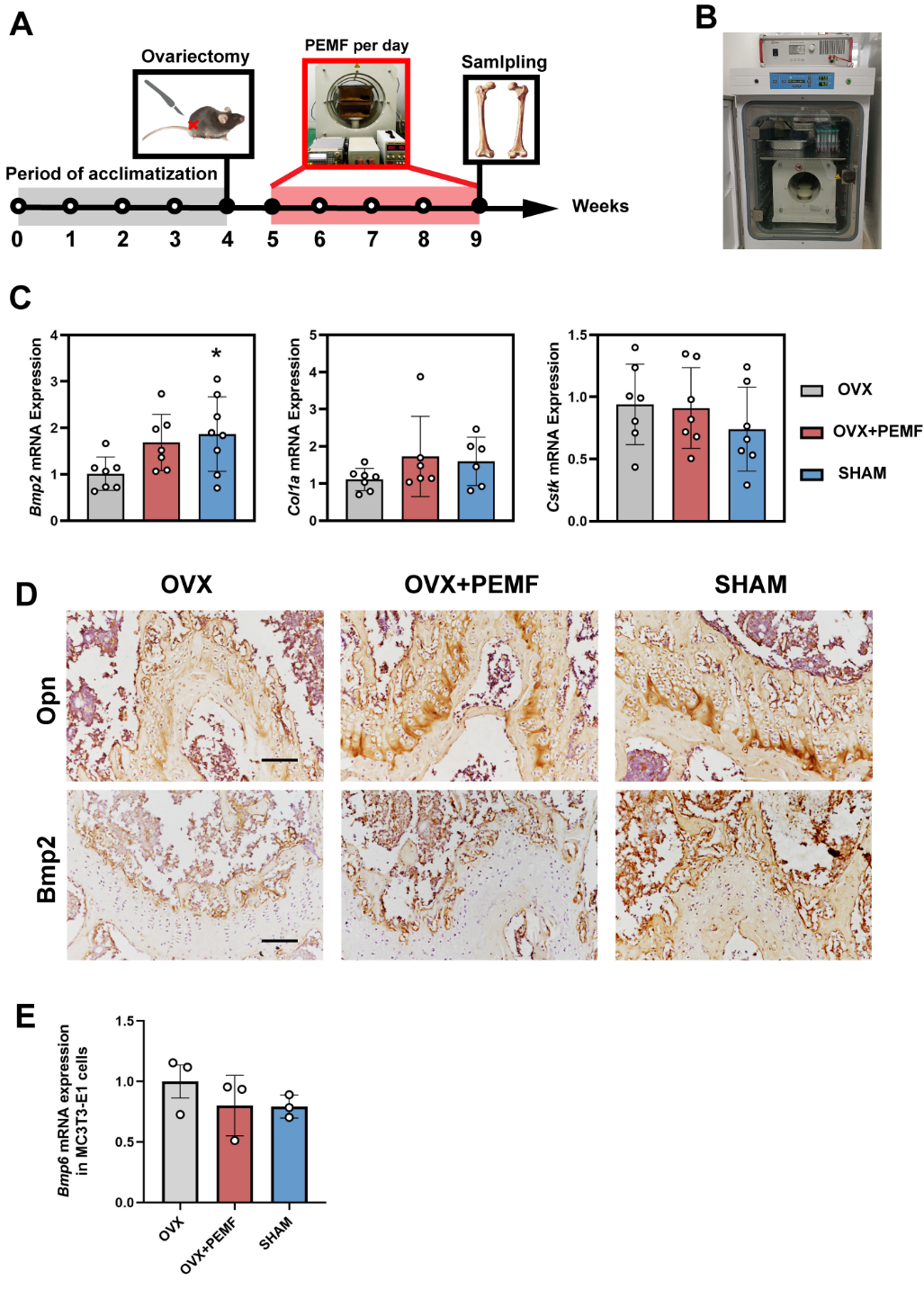


**Supplementary figure 1**  (A) Schematic diagram of the experimental process. (B) The PEMF device for in vitro experiments. (C) Relative mRNA expression of *Bmp2, Col1a,* and *Cstk* detected by PCR (n=6-8 independent experiments). (D) Immunohistochemical images show the relative protein expression of OPN and BMP2 in the metaphysis area in each group (n=3 independent experiments). (E) Relative mRNA expression of *Bmp6* in MC3T3-E1 cells. Scale bar = 100 μm. *P < 0.05, **P < 0.01, ***P < 0.001, ****P < 0.0001; ns: no significance between the indicated groups.


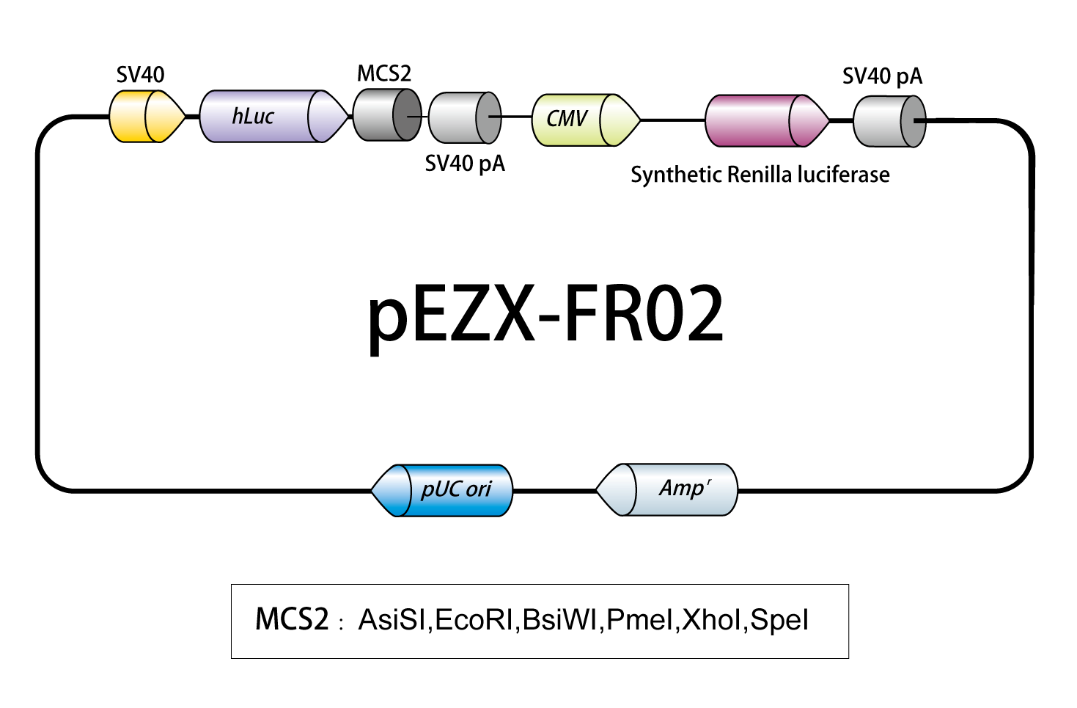


**Supplementary figure 2** Details of pEZX-FR02 plasmid construction.

**3. Database and analysis software used for microRNA sequencing**

| **Database** | **Version** | **Website** |
| --- | --- | --- |
| Mirbase | 21 | http://www.mirbase.org/ftp.shtml |
| Genbank | / | ftp://ftp.ncbi.nlm.nih.gov/genbank/ |
| Rfam | 11.0 | http://rfam.janelia.org/ |
| piRNA | / | http://pirnabank.ibab.ac.in/request.html |
| snoRNABase-human | Version 3 | https://www-snorna.biotoul.fr/index.php |
| Mir2disease | Mar.14, 2011 | http://www.mir2disease.org/ |
| FastQC | v0.11.5 | http://www.bioinformatics.babraham.ac.uk/projects/fastqc/ |
| **Analysis software** | **Version** | **Website** |
| Bowtie | v0.12.9 | http://bowtie-bio.sourceforge.net/index.shtml |
| TopHat2 | v2.1.0 | https://ccb.jhu.edu/software/tophat/index.shtml |
| blast | v2.7.0 | https://blast.ncbi.nlm.nih.gov/Blast.cgi |
| Mirdeep2 | v2.0.0.5 | https://www.mdc-berlin.de/content/mirdeep2-documentation |
| edgeR | v3.16.5 | http://www.bioconductor.org/packages/release/bioc/html/edgeR.html |
| targetscan | v6.0 | http://www.targetscan.org/vert_72/ |
| miRanda | v3.3a | http://www.microrna.org/microrna/home.do |
| RNAhybrid | V2.1.2 | https://bibiserv.cebitec.uni-bielefeld.de/rnahybrid/ |
| GO,KEGG annotation | June 14, 2017; Sep 3, 2016 | http://geneontology.org/; https://www.genome.jp/kegg/ |
